# Circulating Carnitine Levels and Breast Cancer: A Matched Retrospective Case-Control Study

**DOI:** 10.3389/fonc.2022.891619

**Published:** 2022-07-07

**Authors:** Jiayi Zhang, Gang Wu, Hailong Zhu, Fengyuan Yang, Shuman Yang, Ann M. Vuong, Jincheng Li, Demiao Zhu, Yiyan Sun, Wei Tao

**Affiliations:** ^1^ Department of Ultrasonography, The First Affiliated Hospital of Jinzhou Medical University, Jinzhou, China; ^2^ Department of Breast Surgery, The First Affiliated Hospital of Jinzhou Medical University, Jinzhou, China; ^3^ Department of Epidemiology and Biostatistics, School of Public Health, Jilin University, Changchun, China; ^4^ Department of Epidemiology and Biostatistics, School of Public Health, University of Nevada, Las Vegas, NV, United States

**Keywords:** carnitine, breast cancer, women, metabolites, risk assessment

## Abstract

**Introduction:**

Epidemiological studies investigating the association between carnitine and breast cancer are scarce.

**Materials and Methods:**

This 1:1 age-matched retrospective case-control study identified 991 female breast cancer cases and 991 female controls without breast cancer using pathological testing. We used targeted metabolomics technology to measure 16 types of whole blood carnitine compounds, such as free carnitine (C0) and octadecanoylcarnitine (C18).

**Results:**

The average age for cases and controls was approximately 50 ± 8.7 years. After adjusting for covariates, each standard deviation (SD) increase in malonylcarnitine (C3DC; OR 0.91; 95% CI 0.83-1.00), decenoylcarnitine (C10:1; OR 0.87; 95% CI 0.79-0.96), and decadienoylcarnitine (C10:2; OR 0.90; 95% CI 0.82-0.99) level was associated with decreased odds of breast cancer. However, higher butyrylcarnitine (C4) levels were associated with increased odds of breast cancer (OR 1.12; 95% CI 1.02-1.23). No statistically significant relationship was noted between other carnitine compounds and breast cancer. The false discovery rates for C3DC, C4, C10:1 and C10:2 were 0.172, 0.120, 0.064 and 0.139, respectively.

**Conclusions:**

Higher levels of C3DC, C10:1, and C10:2 were protective factors for breast cancer, whereas increased C4 levels were a risk factor for the disease.

## 1 Introduction

Breast cancer remains the most common type of malignancy in females not only in China, but also among other countries in the world (1). The age-standardized incidence rate of breast cancer among females was 28.51 per 100,000 in 2014 in China (2). Females between the ages of 35-69 years are projected to have a higher age-standardized incidence rate for breast cancer in 2021, with a rate of 85 per 100,000 (3).

The major challenges with breast cancer survival are delayed and inaccurate diagnoses, which subsequently affect treatment timing, options, and success rates (4, 5). Identifying specific metabolites contributing to breast cancer development and diagnosis may improve prognosis. Park et al. and Rashed et al. found that plasma metabolites, including l-octanoylcarnitine, 5-oxoproline, hypoxanthine, and docosahexaenoic acid, could be potential biomarkers for diagnosing breast cancer (6, 7). Because metabolites are suggested to be related to the progression of breast cancer, they may be useful for preventing and treating breast cancer (8–11).

Carnitine is an amino acid derivative that is comprised of free carnitine and various forms of short-, medium- and long-chain acylcarnitines in its endogenous form. Carnitine has many metabolic functions (Additional file 1), including stimulating hematopoiesis, inhibiting collagen-induced platelet aggregation, preventing programmed cell death in immune cells, and modulating fatty acid oxidation (12, 13). Carnitine is also able to preserve membrane integrity (14), stabilize the physiological coenzyme A (CoASH)/acetyl-CoA ratio in the mitochondria, and reduce lactate production (12, 15). Carnitine may be involved in the pathogenesis of cancer development. There is evidence suggesting that carnitine-induced fatty acid oxidation plays a critical role in the production of NADH, FADH2, NADPH and ATP, which could contribute to the development of tumors (16–18). Carnitine palmitoyltransferase I (CPTI) was reported to be overexpressed in numerous tumors, suggesting it may play an important role in tumor neovascularization (19).

Although carnitine may be related to breast cancer development, existing epidemiological studies are scarce and those that have examined this potential relationship are limited in their sample size (6, 20). A Turkish case-control study, consisting of 58 breast cancer cases and 30 healthy controls, reported that serum carnitine levels in cases after radiotherapy were lower than in controls (20). Another Korean case-control study of 30 breast cancer cases and 16 healthy controls found that higher plasma l-octanoylcarnitine levels were associated with lower breast cancer risk (6). Thus, we used a 1:1 matched case-control study with a relatively large sample size to examine the association between circulating carnitine levels and breast cancer in females.

## 2. Materials and Methods

### 2.1 Study Setting and Subjects

We recruited participants from The First Affiliated Hospital of Jinzhou Medical University located in Jinzhou, Liaoning, China. This hospital provides medical services to approximately 11.2 million people residing in west Liaoning.

The participants were recruited from the Department of Breast Surgery between November 2015 and September 2020. Eligibility criteria included the following: 1) have a clear diagnosis for either breast cancer or non-breast cancer that was confirmed with a pathological test (Additional file 2); 2) have a valid carnitine measurement; and 3) have complete and valid covariate data. Women with current or past carnitine related treatments (i.e., l-carnitine) were excluded. All controls were individually matched to cases by age ( ± 1 year) at a 1:1 ratio. This research was approved by the Institutional Review Board (IRB; Project #: 202007) at the hospital and written informed consent was obtained from all participants.

### 2.2 Breast Cancer

Pathological stage of diagnosis (21) and the histological tumor grades (22) for breast cancer cases were obtained. We also assessed surrogate subtypes of breast cancer; these included human epidermal growth factor receptor-2 [HER2]-positive, Luminal A-like, Luminal B-like (HER2-positive or negative), and Triple negative (22, 23).

### 2.3 Blood Collection and Processing

Participants were asked to fast for at least 8 hours prior to blood collection. Nurses collected whole blood samples using vacutainer tubes (red cap) in the morning. We used dried blood filter paper to produce dried blood spot samples for carnitine assays. These samples were stored at -80 °C until assays were completed.

### 2.4 Carnitine Measurement

We punched the dried blood spot papers into 3-mm discs (24), which were extracted with ethanol and centrifuged (2 min at 1500g) to collect the supernatant. The supernatant was filtered and moved to 96-well plates. Standard carnitine (catalog number: NSK-A; Cambridge Isotope Laboratory, Tewksbury, MA) solution samples served as quality controls. These plates were dried under 50 °C pure nitrogen flow, incubated with a 1-butanol/acetyl chloride mixture, and dried again at 50°C under pure nitrogen flow. Finally, mobile phase solution (80% acetonitrile aqueous solution) was used to dissolve dried samples, which were measured with liquid chromatography coupled with mass spectrometry (high performance liquid chromatography detector LC-20A [Shimadze, Japan] and tandem mass spectrometry detection system AB Sciex 4000 QTrap [AB Sciex, Framinham MA]); this platform is suggested to be able to sensitively detect biomolecules (25). Absolute concentrations (μmol/L) of each carnitine compound was obtained by using a standard curve. We measured circulating levels of the following carnitine compounds: free carnitine [C0], acetylcarnitine [C2], propionylcarnitine [C3], malonylcarnitine [C3DC], butyrylcarnitine [C4], isovalerylcarnitine [C5], tiglylcarnitine [C5:1], hexanoylcarnitine [C6], octanoylcarnitine [C8], decanoylcarnitine [C10], decenoylcarnitine [C10:1], decadienoylcarnitine [C10:2], dodecanoylcarnitine [C12], myristoylcarnitine [C14], palmitoylcarnitine [C16], and octadecanoylcarnitine [C18].

### 2.5 Ascertainment of Covariates

Demographics (age and body mass index [BMI]), lifestyle factors (smoking and alcohol consumption), and medical history (age at menarche, postmenopausal status, hypertension diagnosis, type 2 diabetes diagnosis, personal history of cancer, family history of cancer, and parity) were considered as covariates. BMI was calculated using the formula: measured weight (kg)/(measured height [m])^2^. An individual was considered to be hypertensive if the measured systolic blood pressure was ≥140 mmHg or if the diastolic blood pressure measurement was ≥90 mmHg (26). Participants were considered to have type 2 diabetes based on standard clinical definitions using results from fasting plasma glucose testing, glucose tolerance testing, and glycated hemoglobin (A1C) testing (27). Information on age at menarche, history of cancer, smoking status, alcohol consumption, family history of cancer, postmenopausal status, and parity (subsequently categorized as 0, 1, 2, and 3+) was extracted from medical records.

### 2.6 Statistical Analysis

Characteristics and carnitine levels were descriptively analyzed for cases and controls. We used conditional logistic regression models to estimate odds ratios (ORs) and 95% confidence intervals (CIs) for the association between one standard deviation (SD) increase in carnitine level and breast cancer. The final conditional logistic regression models were adjusted for postmenopausal status and parity, because only these two factors were significantly associated with breast cancer at alpha = 0.05 under bivariate analysis. To address the issue of multiple testing, we also calculated the false discovery rates (FDRs) for all carnitines.

Carnitine compounds that were statistically significant in the logistic regression analysis were subsequently analyzed with multiple linear regression models to identify whether baseline characteristics are associated with circulating levels in the blood. Subgroup analyses by pathological stage of diagnosis, tumor grades, and surrogate subtypes were also performed with unconditional logistic regression models, in which breast cancer in each subgroup was the dependent variable and each carnitine compound (per 1-SD increase) was the independent variable. These models were adjusted for age, BMI, age at menarche, hypertension diagnosis, type 2 diabetes diagnosis, history of cancer, smoking status, alcohol consumption, family history of cancer, postmenopausal status, and parity. We tested for dose response by pathological stage of diagnosis and tumor grades among cases using multiple linear regression models, whereby continuous measures of carnitine (per 1-SD increase) were the independent variables, and pathological stages of diagnosis and tumor grades were dependent variables. *P* for interaction between surrogate subtypes and carnitine levels were tested using the interaction term (surrogate subtypes* carnitine levels [per 1-SD increase]) among cases in unconditional logistic regression models. All models were adjusted for the same covariates described above. All statistical analyses were performed using SPSS (version: 24.0; SPSS, Chicago, IL) and R (Version 3.5.3, R Foundation for Statistical Computing).

## 3. Results

We identified 991 cases and 991 controls, with a mean age of 50.0 years (SD: 8.7 years) and 49.5 years (SD: 8.7 years), respectively (**Table 1**). Among cases, a total of 332 (33.5%), 481 (48.5%), 164 (16.5%), and 14 (1.4%) had a pathologically confirmed diagnosis stage of I, II, III, and IV, respectively. A majority of the cases had a tumor grade of II (61.5%), followed by grade III (12.9%) and grade 1 (5.5%). Approximately 20% of cases (n=199) did not have information on tumor grade. There were 143 (14.4%), 192 (19.3%), 155 (15.6%), and 471 (47.5%) cases with Triple negative, HER2-positive, Luminal A-like, and Luminal B-like (HER2-positive or negative) surrogate subtypes, respectively. surrogate subtype was not available for 3% of the cases. A majority of the controls (96.5%) had a benign breast lump, while others had either a mastitis (2.1%), a benign accessory breast lump (0.7%), hyperplasia of the mammary glands (0.5%), a benign axillary lump (0.1%), or a lipoma of the breast (0.1%).

**Table 1:** Characteristics and carnitine levels for cases and controls.

Variable	Cases (N=991)	Controls (N=991)	*P*
Age (years)	50.0 (8.7)	49.5 (8.7)	0.217
Body mass index (kg/m^2^)	24.3 (3.5)	24.2 (3.3)	0.553
Age at menarche (years)	15.2 (1.7)	15.1 (1.7)	0.340
Hypertension diagnosis (n, %)	110 (11.1)	130 (13.1)	0.169
Type 2 diabetes diagnosis (n, %)	38 (3.8)	26 (2.6)	0.127
History of cancer (n, %)	34 (3.4)	21 (2.1)	0.075
Smoker (n, %)	20 (2.0)	18 (1.8)	0.743
Alcohol consumption (n, %)	2 (0.2)	2 (0.2)	1.000
Family history of cancer (n, %)	44 (4.4)	49 (4.9)	0.595
Postmenopausal status (n, %)	407 (41.1)	355 (35.8)	0.016
**Parity (n, %)**			0.001
0	53 (5.4)	30 (3.0)	
1	645 (65.1)	670 (67.6)	
2	240 (24.2)	264 (26.6)	
3+	53 (5.4)	27 (2.7)	
Free carnitine (C0, μmol/L)	26.63 (8.00)	26.37 (7.44)	0.464
Acetylcarnitine (C2, μmol/L)	10.14 (4.51)	10.01 (4.26)	0.503
Propionylcarnitine (C3, μmol/L)	1.185 (0.557)	1.170 (0.505)	0.519
Malonylcarnitine (C3DC, μmol/L)	0.052 (0.004)	0.056 (0.005)	0.033
Butyrylcarnitine (C4, μmol/L)	0.156 (0072)	0.149 (0.068)	0.027
Isovalerylcarnitine (C5, μmol/L)	0.132 (0.066)	0.133 (0.064)	0.659
Tiglylcarnitine (C5:1, μmol/L)	0.044 (0.002)	0.045 (0.003)	0.517
Hexanoylcarnitine (C6, μmol/L)	0.064 (0.003)	0.065 (0.003)	0.630
Octanoylcarnitine (C8, μmol/L)	0.066 (0.004)	0.069 (0.005)	0.155
Decanoylcarnitine (C10, μmol/L)	0.077 (0.005)	0.078 (0.006)	0.633
Decenoylcarnitine (C10:1, μmol/L)	0.072 (0.004)	0.080 (0.005)	0.002
Decadienoylcarnitine (C10:2, μmol/L)	0.491 (0.034)	0.527 (0.035)	0.018
Dodecanoylcarnitine (C12, μmol/L)	0.055 (0.004)	0.054 (0.003)	0.702
Myristoylcarnitine (C14, μmol/L)	0.068 (0.003)	0.068 (0.006)	0.639
Palmitoylcarnitine (C16, μmol/L)	0.820 (0.053)	0.800 (0.037)	0.295
Octadecanoylcarnitine (C18, μmol/L)	0.463 (0.018)	0.456 (0.017)	0.397

Unless otherwise specified, variables are presented as the mean (standard deviation). P < 0.05 is considered to be statistically significant.

Postmenopausal status and parity were significantly associated with breast cancer status (**Table 1**). Age, BMI, age at menarche, hypertension diagnosis, type 2 diabetes diagnosis, history of cancer, smoking status, alcohol consumption, and family history of cancer were not significantly different between cases and controls.

**Figure 1:**
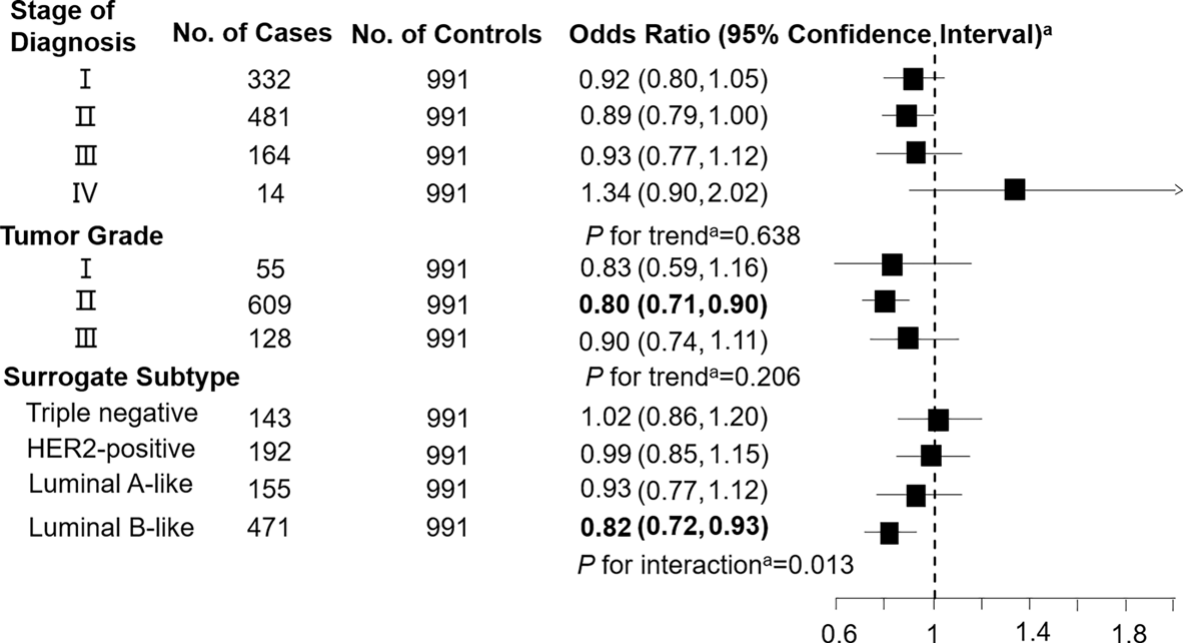
Association between malonylcarnitine (C3DC; per 1-SD increase) and breast cancer by pathological stage of diagnosis, tumor grade, and surrogate subtype. ^a^All models were adjusted for age, body mass index, age at menarche, hypertension diagnosis, type 2 diabetes diagnosis, history of cancer, smoking status, alcohol consumption, family history of cancer, postmenopausal status, and parity. Luminal B-like included HER2-positive and negative. Bold values are statistically significant at α = 0.05.

**Figure 2:**
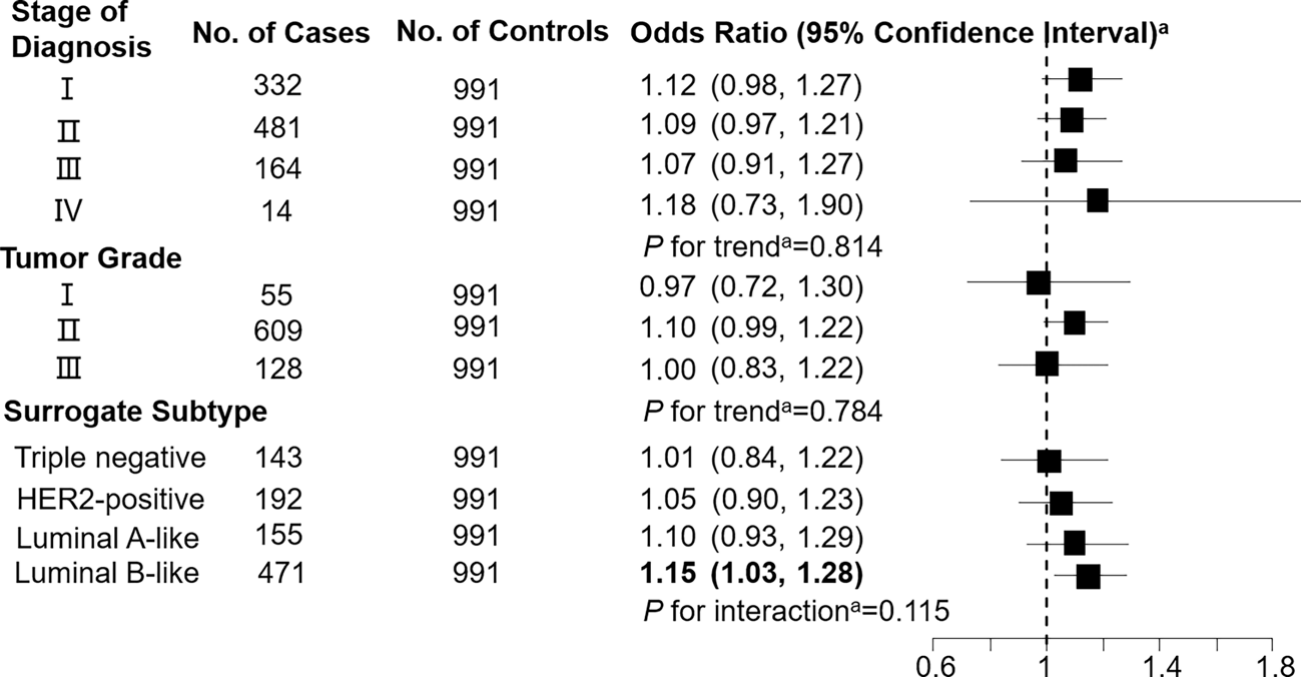
Association between butyrylcarnitine (C4; per 1-SD increase) and breast cancer by pathological stage of diagnosis, tumor grade, and surrogate subtype. ^a^All models were adjusted for age, body mass index, age at menarche, hypertension diagnosis, type 2 diabetes diagnosis, history of cancer, smoking status, alcohol consumption, family history of cancer, postmenopausal status, and parity. Luminal B-like included HER2-positive and negative. Bold values are statistically significant at α = 0.05.

**Figure 3:**
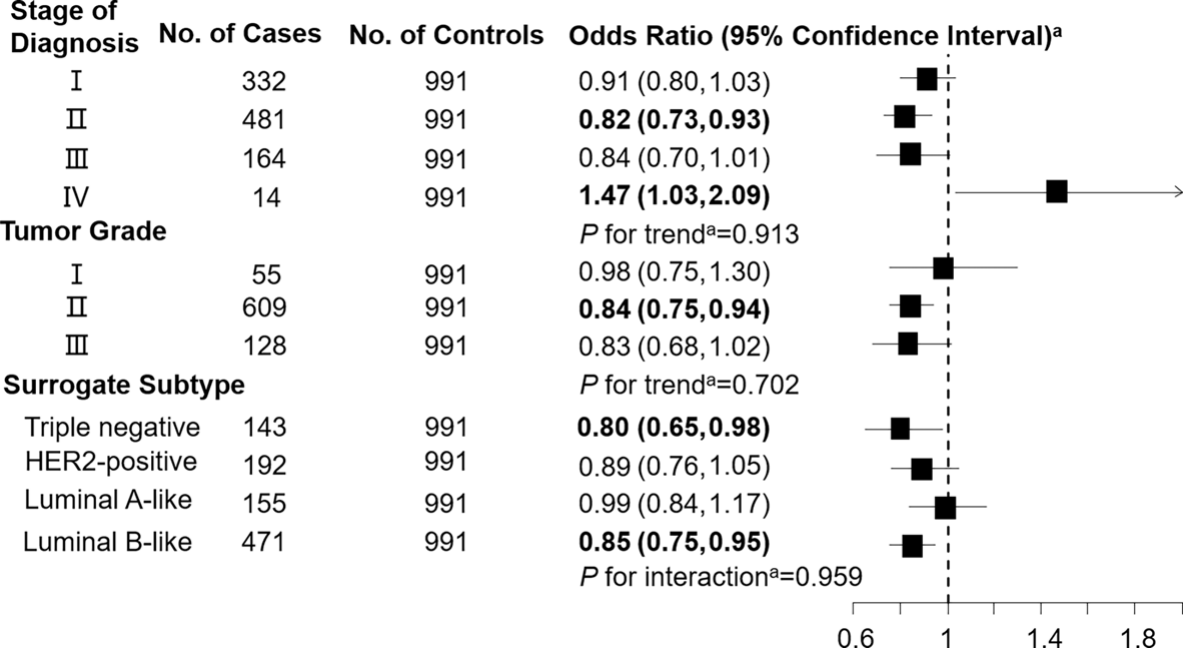
Association between decenoylcarnitine (C10:1; per 1-SD increase) and breast cancer by pathological stage of diagnosis, tumor grade, and surrogate subtype. ^a^All models were adjusted for age, body mass index, age at menarche, hypertension diagnosis, type 2 diabetes diagnosis, history of cancer, smoking status, alcohol consumption, family history of cancer, postmenopausal status, and parity. Luminal B-like included HER2-positive and negative. Bold values are statistically significant at α = 0.05.

**Figure 4:**
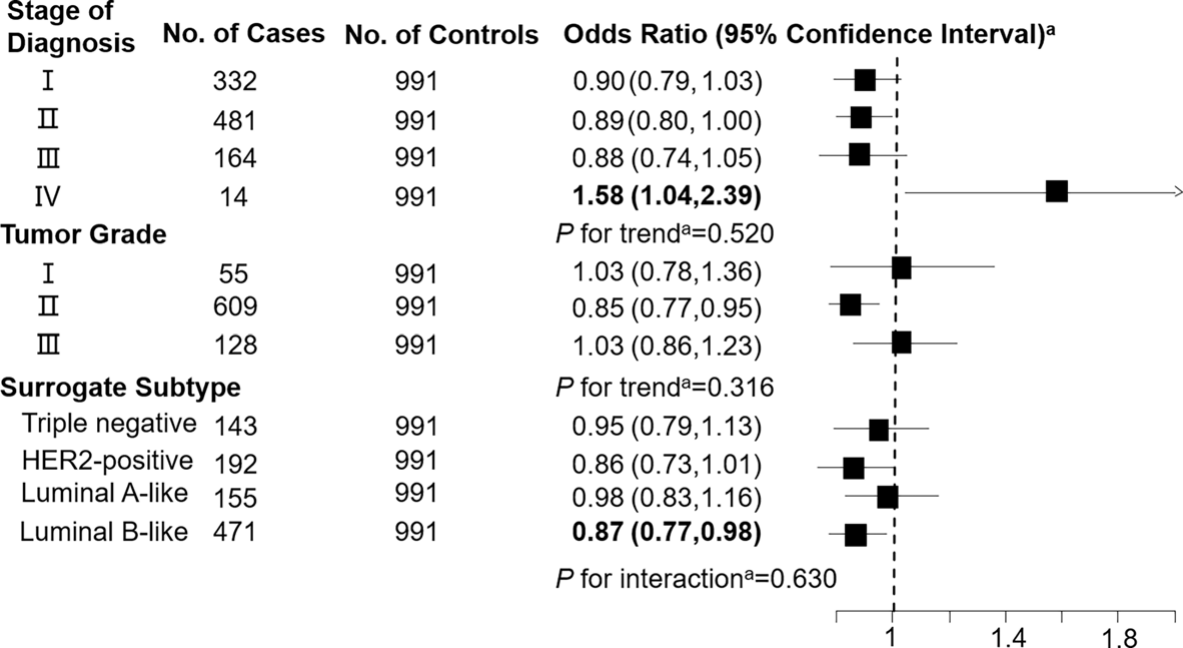
Association between decadienoylcarnitine (C10:2; per 1-SD increase) and breast cancer by pathological stage of diagnosis, tumor grade, and surrogate subtype. ^a^All models were adjusted for age, body mass index, age at menarche, hypertension diagnosis, type 2 diabetes diagnosis, history of cancer, smoking status, alcohol consumption, family history of cancer, postmenopausal status, and parity. Luminal B-like included HER2-positive and negative. Bold values are statistically significant at α = 0.05.

Levels of C3DC, C10:1 and C10:2 in cases were lower than in controls, whereas C4 levels were higher in cases than in controls (**Table 1**). We noted decreased odds of breast cancer with increasing levels of C3DC (OR 0.91; 95% CI 0.83-1.00), C10:1 (OR 0.87; 95% CI 0.79-0.96), and C10:2 (OR 0.90; 95% CI 0.82-0.99) (**Table 2**). However, higher C4 levels were associated increased risk of breast cancer (OR 1.12; 95% CI 1.02-1.23). The FDRs for C3DC, C4, C10:1, and C10:2 were 0.172, 0.120, 0.064, and 0.139, respectively. No other carnitine compounds were associated with breast cancer. The association of C3DC, C4, C10:1, and C10:2 with breast cancer did not differ with stage of diagnosis or tumor grade (all *P* for trend > 0.1; **Figures 1**–**4**). Subgroup analysis by surrogate subtype suggests that the impact of C4 on breast cancer was higher for individuals with the luminal B-like (HER2-positive or negative) subtype (*P* for interaction = 0.013; **Figure 1**). However, we did not observe any evidence to suggest that surrogate subtype modifies the association of C4, C10:1, or C10:2 with breast cancer (**Figures 2**–**4**).

**Table 2:** Multivariable logistic regression analysis^a^ of the association between carnitine levels (per 1-SD increase) and breast cancer.

Carnitine (Abbreviation)	Odds Ratio (95% Confidence Interval)	*P*	False Discovery Rate
Free carnitine (C0)	1.05 (0.96, 1.15)	0.298	0.659
Acetylcarnitine (C2)	1.04 (0.94, 1.16)	0.435	0.659
Propionylcarnitine (C3)	1.03 (0.94, 1.14)	0.494	0.659
Malonylcarnitine (C3DC)	0.91 (0.83, 1.00)	0.043	0.172
Butyrylcarnitine (C4)	1.12 (1.02, 1.23)	0.015	0.120
Isovalerylcarnitine (C5)	0.98 (0.89, 1.08)	0.722	0.825
Tiglylcarnitine (C5:1)	0.98 (0.89, 1.08)	0.680	0.825
Hexanoylcarnitine (C6)	0.99 (0.91, 1.09)	0.880	0.880
Octanoylcarnitine (C8)	0.94 (0.86, 1.03)	0.196	0.627
Decanoylcarnitine (C10)	0.99 (0.90, 1.08)	0.810	0.864
Decenoylcarnitine (C10:1)	0.87 (0.79, 0.96)	0.004	0.064
Decadienoylcarnitine (C10:2)	0.90 (0.82, 0.99)	0.026	0.139
Dodecanoylcarnitine (C12)	1.03 (0.94, 1.13)	0.483	0.659
Myristoylcarnitine (C14)	1.04 (0.94, 1.14)	0.448	0.659
Palmitoylcarnitine (C16)	1.06 (0.95, 1.17)	0.288	0.659
Octadecanoylcarnitine (C18)	1.04 (0.95, 1.14)	0.372	0.659

a Models were adjusted for postmenopausal status and parity. P < 0.05 indicates a significant association between carnitine and breast cancer.

In the multivariable models, C3DC, C4 and C10:1 were positively associated with age among cases (Additional file 3). C3DC was inversely associated with postmenopausal status and parity. C4 was positively associated with BMI, but inversely associated with family history of cancer. However, C10:2 was positively associated with family history of cancer. C10:1 was inversely associated with postmenopausal status. With regard to controls, C3DC and C10:1 were positively associated with parity (Additional file 4). Lastly, C4 was positively associated with age, whereas no relationship was observed between baseline characteristics and C10:2.

## 4. Discussion

This 1:1 individually matched case-control study found that C3DC, C4, C10:1, and C10:2 were significantly associated with increased risk of breast cancer. Stage of diagnosis, tumor grade, and surrogate subtype did not modify the association of C3DC, C10:1, and C10:2 with breast cancer. However, C4 was more strongly associated with breast cancer among individuals with a luminal B-like (HER2-positive or negative) subtype than other surrogate subtypes.

To the best of our knowledge, this is the first large epidemiological study examining the impact of 16 types of carnitine on breast cancer in females. We identified four types of carnitine, including C3DC, C4, C10:1 and C10:2, to be significantly associated with breast cancer, though the directionality of the association differed by the carnitine compound itself. In particular, we observed negative association of C3DC, C10:1, and C10:2 with breast cancer, which is partly consistent with a previous study where serum carnitine levels were lower in cases than in controls after radiotherapy (20). In contrast, a previous case-control study identified l-octanoylcarnitine as a candidate biomarker for breast cancer (6) - a conclusion that does not align with our finding of a null association between C8 and breast cancer. The underlying reasons for this discrepancy remains unclear. However, it may be partially attributed to the differences in the study population (Korean vs. Chinese), sample size (40 cases and 30 controls vs. 991 cases and 991 controls) and design (unmatched case-control study vs. matched case-control study) between the studies.

Given the high prevalence of breast cancer in females, circulating levels of C3DC, C4, C10:1, and C10:2 may have large clinical implications for breast cancer diagnosis as well as potentially risk assessment and treatment. Similar conclusions regarding study implications have also been conveyed by previous studies (6, 7, 9, 10). Further, carnitine levels can be modified *via* the consumption of animal-based products and l-carnitine supplements (28, 29). L-carnitine has been suggested to treat many oxidative stress related conditions, such as heart failure, angina and weight loss (30). Supplementation of specific carnitine compounds (e.g., C3DC, C10:1, and C10:2) can be potential therapeutic and/or prevention strategies for breast cancer following validation of our findings in human trials.

We report inconsistent association between various types of carnitine and breast cancer. The specific biological mechanisms for the relationships identified between C3DC, C4, C10:1, and C10:2 with breast cancer are still uncertain. It is postulated that higher levels of acylcarnitine indicate higher rates of mitochondrial fatty acid oxidation (13), which fuels breast cancer growth (16–18, 31). This mechanism is likely responsible for the positive association between C4 and breast cancer. In contrast, carnitine balances the CoASH/acetyl-CoA ratio (12, 15). A balanced CoASH/acetyl-CoA ratio removes excessive short- and medium-chain fatty acids from the mitochondria, which can potentially be toxic (32). This supports the negative association of C3DC, C10:1, and C10:2 with breast cancer. The physiological mechanism in the pathogenesis of breast cancer is likely to vary by different carnitine compounds (13).

Our study has several strengths. We had a relatively large sample size. Secondly, all cases and controls were confirmed with pathological testing, reducing concerns of outcome misclassification bias. The individually 1:1 matched case-control study design alleviates concerns of potential confounding by age. We further excluded individuals with carnitine related treatments to rule out the influence of the potential medical effects on circulating carnitine levels. We collected fasting blood samples to limit the influence of diet on carnitine measurements. Lastly, all blood samples were drawn in the morning to control for the potential impact of the circadian rhythm on carnitine levels (33).

Our findings should be interpreted within the context of a number of limitations. First, due to the case-control study design, we cannot make a causal inference about the relationship between C3DC, C4, C10:1, or C10:2 with breast cancer. Second, this study did not have data on a number of risk factors that have been identified for breast cancer, such as physical activity, hormone replacement therapy, and diethylstilbestrol use. Potential residual confounding cannot be completely ruled out. Third, a majority of the controls (96.5%) in this study had a benign breast lump. Whether similar conclusions can be drawn using healthy controls is still unclear. Our findings may not be generalizable as we used a hospital-based population, which may be influenced by selection bias. Lastly, we only investigated carnitines in this study, because the targeted approach could only measure a limited set of metabolites and funding was limited (34).

## Conclusions

Higher levels of C3DC, C10:1, and C10:2 were found be protective factors for breast cancer. In contrast, higher C4 levels were identified as a risk factor for breast cancer. These findings substantially expand our understandings about the relationship between carnitine and breast cancer. Whether these carnitine compounds have an impact on breast cancer development requires further examination using high quality prospective cohort studies.

## Data Availability Statement

The datasets generated and/or analysed during the current study are not publicly available due to ethical reasons but are available from the corresponding author on reasonable request.

## Ethics Statement

The studies involving human participants were reviewed and approved by This research was approved by the Institutional Review Board (IRB; Project #: 202007) at The First Affiliated Hospital of Jinzhou Medical University. The patients/participants provided their written informed consent to participate in this study.

## Author Contributions

Conception, design, and analysis (WT, SY and JZ). Interpretation of data (all authors). Drafting the article (JZ). Critically revising the article for important intellectual content (all authors). Final approval of the version to be published (all authors), and agreement to be accountable for all aspects of the work (all authors).

## Funding

This work is supported by a research grant from the Jinzhou Science and Technology Bureau (Grant Number: JZ2022B039).

## Conflict of Interest

The authors declare that the research was conducted in the absence of any commercial or financial relationships that could be construed as a potential conflict of interest.

## Publisher’s Note

All claims expressed in this article are solely those of the authors and do not necessarily represent those of their affiliated organizations, or those of the publisher, the editors and the reviewers. Any product that may be evaluated in this article, or claim that may be made by its manufacturer, is not guaranteed or endorsed by the publisher.

## Supplementary Material

The Supplementary Material for this article can be found onlineat: https://www.frontiersin.org/articles/10.3389/fonc.2022.891619/full#supplementary-material
