# Supplementary material for: Circulating Carnitine Levels and Breast Cancer: A Matched Retrospective Case-Control Study
Source: Front Oncol. 2022 Jul 7;12:891619. doi: 10.3389/fonc.2022.891619 (PMC9300951; doi:10.3389/fonc.2022.891619)
Supplement: Supplementary file 1 [file DataSheet_1.docx]

**Additional file 1. Clinical functions of carnitine**

| **Function** | **Reference** |
| --- | --- |
| Stimulation of hematopoiesis, inhibition of collagen-induced platelet aggregation, prevention of programmed cell death in immune cells, and modulation of fatty acid and whole-body fat oxidation | 1. Karlic H, Lohninger A. Supplementation of L-carnitine in athletes: does it make sense? Nutrition. 2004 Jul-Aug;20(7-8):709-15. Epub 2004/06/24. doi:10.1016/j.nut.2004.04.003. Cited in: Pubmed; PMID 15212755. 2. Wolf M, Chen S, Zhao X, Scheler M, Irmler M, Staiger H, Beckers J, de Angelis MH, Fritsche A, Haring HU, Schleicher ED, Xu G, Lehmann R, Weigert C. Production and release of acylcarnitines by primary myotubes reflect the differences in fasting fat oxidation of the donors. J Clin Endocrinol Metab. 2013 Jun;98(6):E1137-42. doi:10.1210/jc.2012-3976. Cited in: Pubmed; PMID 23633211. |
| Regulation of muscle bioenergetics | 1. Friolet R, Hoppeler H, Krahenbuhl S. Relationship between the coenzyme A and the carnitine pools in human skeletal muscle at rest and after exhaustive exercise under normoxic and acutely hypoxic conditions. J Clin Invest. 1994 Oct;94(4):1490-5. Epub 1994/10/01. doi:10.1172/JCI117488. Cited in: Pubmed; PMID 7929825. 2. Gnoni A, Longo S, Gnoni GV, Giudetti AM. Carnitine in Human Muscle Bioenergetics: Can Carnitine Supplementation Improve Physical Exercise? Molecules. 2020 Jan 1;25(1). Epub 2020/01/08. doi:10.3390/molecules25010182. Cited in: Pubmed; PMID 31906370. |
| Improvement of antioxidant system and prevention of radiation-induced adverse effects | 1. Pekala J, Patkowska-Sokola B, Bodkowski R, Jamroz D, Nowakowski P, Lochynski S, Librowski T. L-carnitine--metabolic functions and meaning in humans life. Curr Drug Metab. 2011 Sep;12(7):667-78. doi:10.2174/138920011796504536. Cited in: Pubmed; PMID 21561431. 2. Ozmen HK, Erdemci B, Askin S, Sezen O. Carnitine and Adiponectin Levels in Breast Cancer after Radiotherapy. Open Med (Wars). 2017;12:189-194. Epub 2017/07/22. doi:10.1515/med-2017-0028. Cited in: Pubmed; PMID 28730178. |
| Preservation of membrane integrity | Siliprandi N, Di Lisa F, Menabo R. Clinical use of carnitine. Past, present and future. Adv Exp Med Biol. 1990;272:175-81. Epub 1990/01/01. doi:10.1007/978-1-4684-5826-8_11. Cited in: Pubmed; PMID 2103685. |
| Maintaining normal mitochondrial function and reduction of lactate production | 1. Friolet R, Hoppeler H, Krahenbuhl S. Relationship between the coenzyme A and the carnitine pools in human skeletal muscle at rest and after exhaustive exercise under normoxic and acutely hypoxic conditions. J Clin Invest. 1994 Oct;94(4):1490-5. Epub 1994/10/01. doi:10.1172/JCI117488. Cited in: Pubmed; PMID 7929825. 2. Karlic H, Lohninger A. Supplementation of L-carnitine in athletes: does it make sense? Nutrition. 2004 Jul-Aug;20(7-8):709-15. Epub 2004/06/24. doi:10.1016/j.nut.2004.04.003. Cited in: Pubmed; PMID 15212755. 3. Reuter SE, Evans AM. Carnitine and acylcarnitines: pharmacokinetic, pharmacological and clinical aspects. Clin Pharmacokinet. 2012 Sep 1;51(9):553-72. Epub 2012/07/19. doi:10.2165/11633940-000000000-0000010.1007/BF03261931. Cited in: Pubmed; PMID 22804748. |
| Regulation of the tumor growth | 1. Carracedo A, Cantley LC, Pandolfi PP. Cancer metabolism: fatty acid oxidation in the limelight. Nat Rev Cancer. 2013 Apr;13(4):227-32. Epub 2013/03/01. doi:10.1038/nrc3483. Cited in: Pubmed; PMID 23446547. 2. Hossain F, Al-Khami AA, Wyczechowska D, Hernandez C, Zheng L, Reiss K, Valle LD, Trillo-Tinoco J, Maj T, Zou W, Rodriguez PC, Ochoa AC. Inhibition of Fatty Acid Oxidation Modulates Immunosuppressive Functions of Myeloid-Derived Suppressor Cells and Enhances Cancer Therapies. Cancer Immunol Res. 2015 Nov;3(11):1236-47. Epub 2015/05/31. doi:10.1158/2326-6066.CIR-15-0036. Cited in: Pubmed; PMID 26025381. 3. Ricciardi MR, Mirabilii S, Allegretti M, Licchetta R, Calarco A, Torrisi MR, Foa R, Nicolai R, Peluso G, Tafuri A. Targeting the leukemia cell metabolism by the CPT1a inhibition: functional preclinical effects in leukemias. Blood. 2015 Oct 15;126(16):1925-9. Epub 2015/08/16. doi:10.1182/blood-2014-12-617498. Cited in: Pubmed; PMID 26276667. 4. Qu Q, Zeng F, Liu X, Wang QJ, Deng F. Fatty acid oxidation and carnitine palmitoyltransferase I: emerging therapeutic targets in cancer. Cell Death Dis. 2016 May 19;7:e2226. Epub 2016/05/20. doi:10.1038/cddis.2016.132. Cited in: Pubmed; PMID 27195673. 5. Marx W, Teleni L, Opie RS, Kelly J, Marshall S, Itsiopoulos C, Isenring E. Efficacy and Effectiveness of Carnitine Supplementation for Cancer-Related Fatigue: A Systematic Literature Review and Meta-Analysis. Nutrients. 2017 Nov 7;9(11). Epub 2017/11/08. doi:10.3390/nu9111224. Cited in: Pubmed; PMID 29112178. 6. Melone MAB, Valentino A, Margarucci S, Galderisi U, Giordano A, Peluso G. The carnitine system and cancer metabolic plasticity. Cell Death Dis. 2018 Feb 14;9(2):228. Epub 2018/02/16. doi:10.1038/s41419-018-0313-7. Cited in: Pubmed; PMID 29445084. |

**Additional file 2. Typical pathological pictures for confirming cases and controls**

**
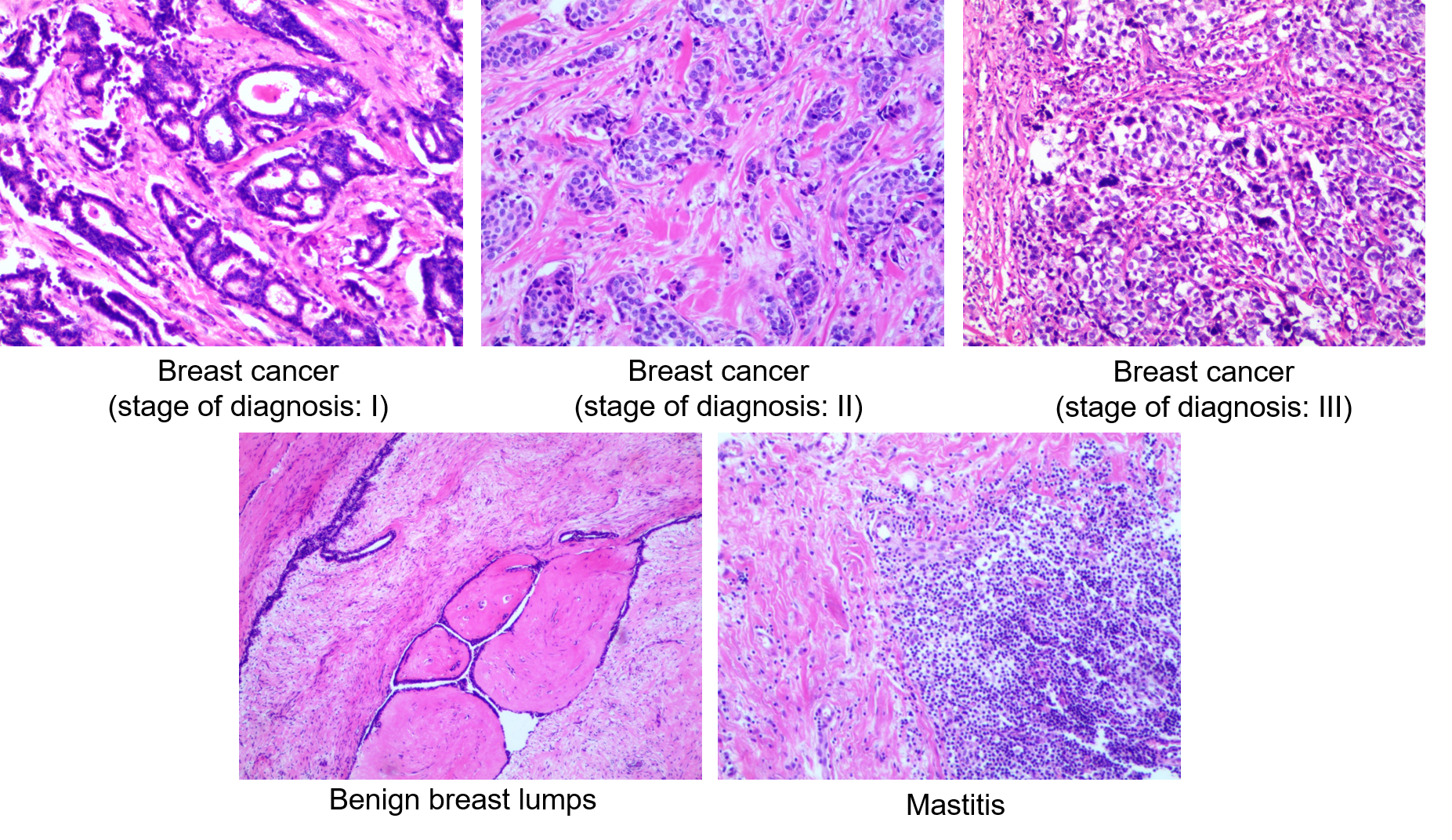
**

**Additional file 3. Association of baseline characteristics with malonylcarnitine (C3DC), butyrylcarnitine (C4), decenoylcarnitine (C10:1), and decadienoylcarnitine (C10:2) in cases**

| **Characteristic** | **Unit of Change or Comparison** | **C3DC**  β (*P*) | **C4**  β (*P*) | **C10:1**  β (*P*) | **C10:2**  β (*P*) |
| --- | --- | --- | --- | --- | --- |
| Age (years) | Per 1-SD increase | 0.006 (0.005) | 0.007 (0.037) | 0.007 (0.001) | 0.026 (0.086) |
| Body mass index | Per 1-SD increase | 0.002 (0.185) | 0.005 (0.020) | 0.001 (0.509) | 0.017 (0.109) |
| Age at menarche | Per 1-SD increase | -0.001 (0.585) | 0.0004 (0.873) | -0.0002 (0.925) | -0.005 (0.678) |
| Hypertension diagnosis | Yes vs. no | 0.004 (0.373) | 0.012 (0.136) | 0.009 (0.091) | 0.054 (0.142) |
| Type 2 diabetes  diagnosis | Yes vs. no | -0.013 (0.091) | 0.006 (0.655) | 0.019 (0.020) | 0.046 (0.433) |
| History of cancer | Yes vs. no | 0.008 (0.293) | 0.002 (0.902) | -0.007 (0.432) | 0.108 (0.068) |
| Smoker | Yes vs. no | -0.006 (0.551) | -0.027 (0.093) | -0.002 (0.831) | 0.042 (0.589) |
| Family history of  cancer | Yes vs. no | 0.005 (0.466) | -0.024 (0.029) | 0.010 (0.159) | 0.122 (0.019) |
| Postmenopausal status | Yes vs. no | -0.008 (0.043) | 0.009 (0.129) | -0.011 (0.006) | 0.005 (0.855) |
| Parity |  |  |  |  |  |
|  | 1 vs. 0 | -0.010 (0.107) | 0.015 (0.145) | 0.005 (0.465) | -0.037 (0.440) |
|  | 2 vs. 0 | -0.018 (0.008) | 0.013 (0.228) | -0.001 (0.890) | -0.043 (0.403) |
|  | 3+ vs. 0 | -0.017 (0.045) | 0.006 (0.660) | -0.004 (0.664) | -0.106 (0.111) |

Models were adjusted for age, body mass index, age at menarche, hypertension diagnosis, type 2 diabetes diagnosis, history of cancer, smoking status, alcohol consumption, family history of cancer, postmenopausal status, and parity.

**Additional file 4.** **Association of baseline characteristics with malonylcarnitine (C3DC), butyrylcarnitine (C4), decenoylcarnitine (C10:1), and decadienoylcarnitine (C10:2) in controls**

| **Characteristic** | **Unit of Change or Comparison** | **C3DC**  β (*P*) | **C4**  β (*P*) | **C10:1**  β (*P*) | **C10:2**  β (*P*) |
| --- | --- | --- | --- | --- | --- |
| Age (years) | Per 1-SD increase | -0.001 (0.659) | 0.007 (0.018) | -0.001 (0.838) | 0.007 (0.968) |
| Body mass index | Per 1-SD increase | -0.001 (0.520) | 0.001 (0.661) | -0.003 (0.086) | -0.007 (0.571) |
| Age at menarche | Per 1-SD increase | 0.001 (0.377) | -0.004 (0.110) | 0.001 (0.930) | -0.001 (0.973) |
| Hypertension diagnosis | Yes vs. no | 0.003 (0.509) | 0.001 (0.985) | 0.001 (0.817) | 0.011 (0.753) |
| Type 2 diabetes  diagnosis | Yes vs. no | -0.001 (0.874) | 0.004 (0.793) | 0.015 (0.169) | 0.053 (0.460) |
| History of cancer | Yes vs. no | -0.013 (0.203) | 0.024 (0.108) | -0.009 (0.479) | -0.015 (0.849) |
| Smoker | Yes vs. no | 0.001 (0.932) | 0.003 (0.841) | 0.013 (0.303) | 0.130 (0.124) |
| Family history of  cancer | Yes vs. no | 0.006 (0.348) | -0.019 (0.054) | -0.006 (0.445) | -0.002 (0.962) |
| Postmenopausal status | Yes vs. no | 0.001 (0.948) | 0.010 (0.109) | 0.002 (0.632) | 0.001 (0.964) |
| Parity |  |  |  |  |  |
|  | 1 vs. 0 | 0.020 (0.015) | -0.006 (0.636) | 0.028 (0.006) | 0.115 (0.083) |
|  | 2 vs. 0 | 0.021 (0.015) | 0.002 (0.888) | 0.027 (0.010) | 0.144 (0.035) |
|  | 3+ vs. 0 | 0.030 (0.014) | -0.012 (0.525) | 0.046 (0.002) | 0.113 (0.243) |

Models were adjusted for age, body mass index, age at menarche, hypertension diagnosis, type 2 diabetes diagnosis, history of cancer, smoking status, alcohol consumption, family history of cancer, postmenopausal status, and parity.
